# Liang-Ge-San, a classic traditional Chinese medicine formula, protects against lipopolysaccharide-induced inflammation through cholinergic anti-inflammatory pathway

**DOI:** 10.18632/oncotarget.8452

**Published:** 2016-03-28

**Authors:** Jun-Shan Liu, Xi-Duan Wei, Zi-Bin Lu, Pei Xie, Hong-Ling Zhou, Yu-Yao Chen, Jia-Mei Ma, Lin-Zhong Yu

**Affiliations:** ^1^ Department of Traditional Chinese Medicine, Southern Medical University, Guangzhou, P. R. China

**Keywords:** Liang-Ge-San, inflammation, α7 nicotinic cholinergic receptor, NF-κB, Immunology and Microbiology Section, Immune response, Immunity

## Abstract

Liang-Ge-San (LGS) is a classic formula in traditional Chinese medicine, which is widely used to treat acute lung injury (ALI), pharyngitis and amygdalitis in clinic. However, the underlying mechanisms remain poorly defined. In this study, we discovered that LGS exerted potent anti-inflammatory effects in lipopolysaccharide (LPS)-induced inflammation. We found that LGS significantly depressed the production of IL-6 and TNF-α in LPS-stimulated RAW 264.7 macrophage cells. The degradation and phosphorylation of IκBα and the nuclear translocation of NF-κB p65 were also inhibited. Moreover, LGS activated α7 nicotinic cholinergic receptor (α7nAchR). The blockage of α7nAchR by selective inhibitor methyllycaconitine (MLA) or α7nAchR siRNA attenuated the inhibitory effects of LGS on IκBα, NF-κB p65, IL-6 and TNF-α. Critically, LGS significantly inhibited inflammation in LPS-induced ALI rats through the activation of NF-κB signaling pathway. However, these protective effects could be counteracted by the treatment of MLA. Taken together, we first demonstrated anti-inflammatory effects of LGS both *in vitro* and *in vivo* through cholinergic anti-inflammatory pathway. The study provides a rationale for the clinical application of LGS as an anti-inflammatory agent and supports the critical role of cholinergic anti-inflammatory pathway in inflammation.

## INTRODUCTION

The high mortality human diseases, such as acute lung injury (ALI), sepsis and shock, are caused by excessive inflammation [1-3]. Innate immune responses to the inflammation have been well delineated by humoral factors. However, recent research indicated that cholinergic anti-inflammatory pathway could significantly suppress peripheral inflammatory responses [4]. It potentially originates from activating the vagus nerve to release acetylcholine (Ach), which binds to the α7 cholinergic receptor (α7nAchR) on immune cells and then suppresses the production of inflammatory cytokines, including interleukin-6 (IL-6) and tumor necrosis factor (TNF-α), high-mobility group box 1 proteins and matrix metalloproteinase 9 [5-9]. Studies have demonstrated that stimulating the vagus nerve could diminish the activation of macrophages in lethal sepsis rats and the cholinergic agonist nicotine could improve their survivals [10, 11]. It has been also reported that the activation of cholinergic anti-inflammatory pathway could reduce ALI. α7nAchR agonist (nicotine, choline and PNU-282987) can suppress the excess lung water, reduce inflammatory cells, myeloperoxidase and proteins in the bronchoalveolar lavage fluid (BALF), and down-regulate pro-inflammatory chemokines and cytokines, including IL-6, TNF-α, macrophage inflammatory protein-1α (MIP-1α) and macrophage inflammatory protein-2 (MIP-2) in LPS or acid-induced ALI murine model [12, 13].

Liang-Ge-San (LGS) is a classic Chinese medicine formula. This decoction was first described in “Taiping Huimin Heji Jufang”, the pharmacopeia in Song dynasty. There are seven herbs, including *Forsythiasuspense*, *Rheum palmatum*, *Scutellaria baicalensis*, *Gardenia jasminoides*, *Glycyrrhiza uralensis*, *Mentha haplocalyx* and *Natrii Sulfas* in the formula. It has been widely used for centuries to clear heat and fire in traditional Chinese medicine. Nowadays, LGS exhibits curative effects on ALI, pharyngitis, amygdalitis and pneumonia [14, 15]. In previous studies, we found that LGS could significantly inhibit the inflammation in LPS-induced ALI rats through suppressing the rise of wet-to-dry ratio of lung tissue and lung permeability. It could also decrease the helper T-cells (Th1-to-Th2) ratio in both of peripheral blood and BALF [16, 17]. However, the underlying mechanisms remain poorly defined.

In this study, we first found that LGS exerted anti-inflammatory effects *via* activating cholinergic anti-inflammatory pathway, which led to the inhibition of NF-κB pathway *in vitro* and *in vivo*. Our research provided scientific evidence for the therapeutic application of LGS and supported the critical role of cholinergic anti-inflammatory pathway in inflammation.

## RESULTS

### Cytotoxicity of LGS in RAW 264.7 macrophage cells

**Figure 1:**
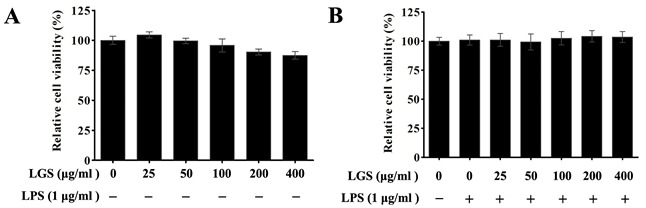
The cytotoxicity of LGS with or without LPS in RAW 264.7 macrophage cells The viability was determined by MTT assay. **A.** Macrophages were incubated with LGS (25 ~ 400 μg/ml) alone for 24 h. **B.** Macrophages were treated with LGS at different concentrations in the presence of LPS (1 μg/ml) for 24 h. Data are represent as percentage of the cell viability as mean ± S.D. of three independent experiments.

MTT assay was used to investigate the cytotoxicity of LGS in RAW 264.7 cells. Results showed that LGS (25 ~ 400 μg/ml) had no obvious effects on cell viability after 24 h treatment (Figure 1A). Furthermore, we evaluated the cell viability of LGS with LPS (1 μg/ml). It was found that both of LGS and LPS treatments didn't show significant cytotoxicity in RAW 264.7 cells (Figure 1B). Therefore, LGS at the concentrations of 25 ~ 400 μg/ml was selected in the subsequent experiments.

### LGS inhibits the release of IL-6, TNF-α in RAW 264.7 macrophage cells

**Figure 2:**
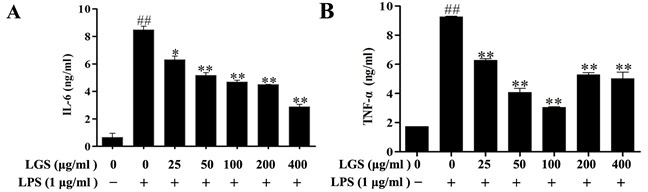
LGS attenuates the production of IL-6 and TNF-α in LPS-stimulated RAW 264.7 cells RAW 264.7 cells were cultured with indicated concentrations of LGS in the presence or absence of LPS (1 μg/ml) for 24 h. Quantitative analyses of IL-6 **A.** and TNF-α **B.** in supernatants of macrophages were measured by ELISA. ^##^*P* < 0.01 *versus* control, *P < 0.05 and **P < 0.01 *versus* LPS treatment, one-way ANOVA, *post hoc* comparisons, Turkey, Cloumns, mean; error bar, S.D.

LPS-treated RAW 264.7 macrophage cells can produce pro-inflammatory cytokines IL-6 and TNF-α, which contribute to inflammation [18, 19]. Hence, we detected the expressions of these cytokines. As shown in Figure 2, resting RAW 264.7 cells released little IL-6 (< 0.2 ng/ml) and TNF-α (< 1 ng/ml). After stimulated by LPS, IL-6 (> 4.2 ng/ml) and TNF-α (> 4.5 ng/ml) were significantly released from RAW 264.7 cells which were inhibited in the presence of LGS (Figure 2A & 2B). It should be noticed that the inhibition of TNF-α was slightly attenuated when the concentration of LGS was exceeded 100 μg/ml (Figure 2B). This phenomenon might result from the hypo-toxic effect of LGS on RAW 264.7 cells. Collectively, these results suggest that LGS can inhibit inflammatory effects in LPS-stimulated RAW 264.7 macrophage cells.

### LGS attenuates NF-κB activation in LPS-stimulated RAW 264.7 macrophage cells

**Figure 3:**
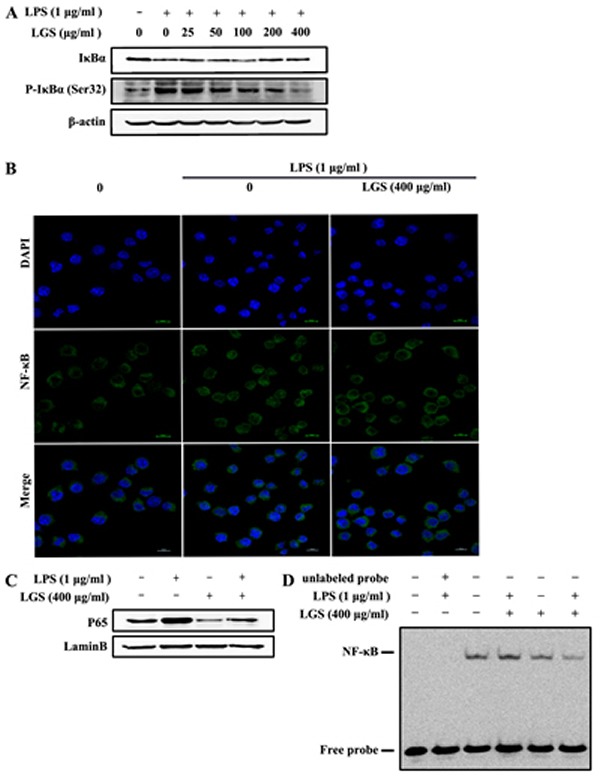
LGS inhibits the activation of NF-κB signaling in LPS-stimulated RAW 264.7 cells **A.** LGS suppresses LPS-induced degradation and phosphorylation of IκBα. RAW 264.7 cells were stimulated with LPS (1 μg/ml) for 30 min and then treated with different concentrations of LGS for 24 h. The expression levels of IκBα and p-IκBα (Ser32) were examined by western blotting. **B.-D.** LGS blocks NF-κB p65 translocation from cytoplasm to nuclear. RAW 264.7 cells were stimulated with LPS (1 μg/ml) for 2 h, incubated with or without LGS (400 μg/ml) for 24 h and observed by confocal microscopy. (200 ×) (B). Expression of NF-κB p65 in nuclear was detected by western blotting (C) and NF-κB nuclear binding activity was measured by EMSA (D).

In inflammatory responses, NF-κB is a critical transcriptional regulator for inflammatory-related gene coding [20]. Therefore, we hypothesized that the anti-inflammatory effects of LGS might be associated with NF-κB pathway. To confirm this hypothesis, we observed the nuclear translocation of NF-κB p65 and the expression of IκBα, whose degradation and phosphorylation makes NF-κB p65 release and transfer into the nuclear [21]. Our results showed that LPS treatment induced the degradation and phosphorylation of IκBα in RAW 264.7 cells, while LGS treatment prevented this phenomenon (Figure 3A). In addition, we found that NF-κB p65 mainly existed in the cytoplasm of RAW 264.7 cells, which was transferred into nuclear after LPS stimulation. Notably, this migration was blocked in the presence of LGS (400 μg/ml) (Figure 3B). Finally, we confirmed the nuclear translocation of NF-κB p65 by western blotting and EMSA, revealing that LGS suppressed endogenous NF-κB p65 nuclear translocation in LPS-stimulated RAW 264.7 cells (Figure 3C & 3D). Taken together, these findings demonstrate that LGS attenuates inflammation by NF-κB signaling pathway.

### LGS suppresses the inflammation and NF-κB pathway via the cholinergic anti-inflammatory pathway

**Figure 4:**
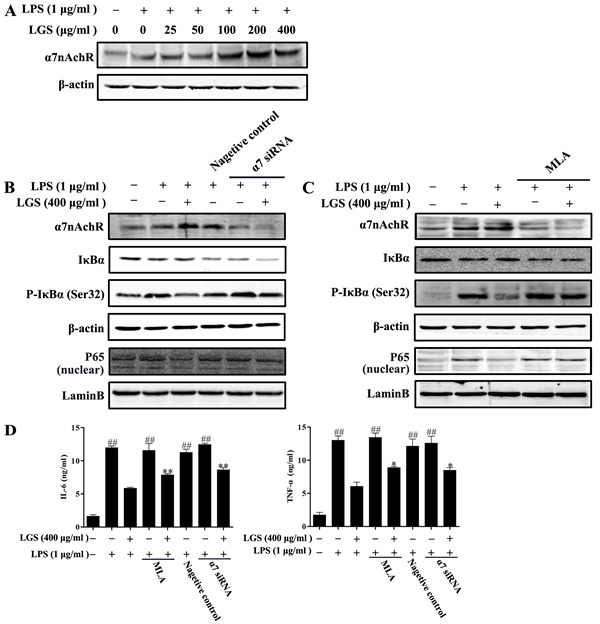
LGS suppresses inflammation and NF-κB pathway *via* α7nAchR activation **A.** LGS increases the expression of α7nAchR. RAW 264.7 cells were pretreated with indicated concentrations of LGS for 24 h and then stimulated with LPS (1 μg/ml) for 2 h. The level of α7nAchR in total lysate was evaluated by western blotting. **B.-C.** LGS suppresses NF-κB pathway *via* α7nAchR. RAW 264.7 cells were transfected with α7nAchR siRNA (120 nM) for 24 h (B) or treated with methyllycaconitine (MLA, 20 μM) for 2 h (C) before cultured with 400 μg/ml LGS for 24 h and then stimulated with LPS (1 μg/ml) for 30 min or 2 h. The levels of α7nAchR (for 2 h), IκBα and p-IκBα (Ser32) (for 30 min) in total lysate and the expression of NF-κB in nuclear were detected by western blotting. **D.** LGS inhibits the production of IL-6 and TNF-α *via* α7nAchR. RAW 264.7 cells were transfected with α7nAchR siRNA (120 nM) for 24 h or pretreated with MLA (20 μM) for 2 h before culturing with LGS (400 μg/ml) in the presence or absence of LPS (1 μg/ml) for 24 h. IL-6 (left panel) and TNF-α (right panel) in the supernatants were determined by ELISA. ^##^*P* < 0.01 *versus* control,*P < 0.05 and **P < 0.01 *versus* LGS and LPS treatment, one-way ANOVA, *post hoc* comparisons, Turkey, Cloumns, mean; error bar, S.D.

α7nAchR expresses on macrophages and plays an important role in cholinergic anti-inflammatory pathway[9]. Previous studies have indicated that nicotine decreased the release of IL-6 and TNF-α *via* α7nAchR in microphages [5, 22]. Therefore, we detected that whether LGS could affect the expression of α7nAchR. As shown in Figure 4A, LGS increased the expression level of α7nAchR in RAW 264.7 cells by western blotting, suggesting that LGS could activate the cholinergic anti-inflammatory pathway.

We further explored the role of α7nAchR in anti-inflammatory effects of LGS. Special α7nAchR siRNA (Figure 4B) or methyllycaconitine (MLA), a selective α7nAchR inhibitor (Figure 4C) was used to abrogate this protein. The results showed that the decrease of α7nAchR counteracted the inhibitory effects of LGS on the degradation and phosphorylation of IκBα and the nuclear translocation of NF-κB in LPS-stimulated RAW 264.7 cells. Moreover, the inhibition of α7nAchR could also decrease the suppressive effects of LGS on the levels of IL-6 and TNF-α (Figure 4D). These data demonstrate that LGS inhibits inflammation and NF-κB pathway which is associated with the activation of cholinergic anti-inflammatory pathway.

### LGS attenuates inflammation in ALI rats through cholinergic anti-inflammatory pathway

**Figure 5:**
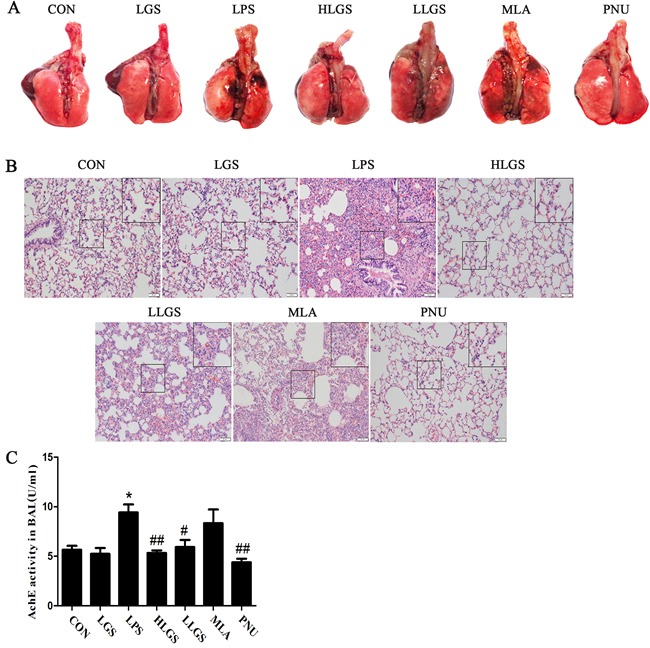
LGS attenuates LPS-induced acute lung injury (ALI) through cholinergic anti-inflammatory pathway LGS with or without methyllycaconitine (MLA, a α7nAchR selective inhibitor) were treated with LPS-induced ALI rats. PNU282987 (PNU), a highly selective AChR α7 agonist, was used as a positive control. **A.** LGS decreases hyperaemia and swelling on the pulmonary surface in LPS-induced ALI rats which can be counteracted in the appearance of MLA. At 8 h after ALI, the rats were sacrificed and the representative lungs removed were photographed. **B.** MLA attenuates the influence of LGS on lung histopathological changes in LPS-induced ALI rats. Lungs were fixated. After paraffin embedding and sectioning at 5 μm thickness, lung tissues were stained by hematoxilin and eosin (H&E) staining. (200 ×) C. MLA counteracts the inhibitory effects of LGS on Acetyl cholinesterase (AChE) in LPS-induced ALI rats. At 8 h after ALI, the rats were sacrificed and the bronchoalveolar lavage fluid (BALF) was obtained. The activity of AChE in supernatants of BALF samples was measured using acetyl cholinesterase kit according to the manufacturer's protocol. *P<0.05 *versus* control, ^#^*P*<0.05 and ^##^*P*<0.01 *versus* LPS, one-way ANOVA, *post hoc* comparisons, Turkey, Cloumns, mean; error bar, S.D.

Previous studies have elucidated that nicotine could reduce inflammatory response in LPS-induced ALI murine model and LGS could protect against LPS-induced ALI rats [12, 16]. Therefore, we detected that whether LGS could prevent ALI through cholinergic anti-inflammatory pathway *in vivo*. As shown in Figure 5A, LGS decreased hyperaemia and swelling on the pulmonary surface in LPS-induced ALI rats dose-dependently. The same phenomena were also observed in the species after a selective α7nAChR agonist PNU282987 (PNU) treatment. It should be pointed out that the characteristic of the pulmonary surface after HLGS treatment was like that after PNU treatment, implying the significant protective activity of LGS. However, the anti-ALI effects of LGS can be counteracted in the presence of MLA. Lung pathologic observation also showed that MLA suppressed the ameliorated effects of LGS on edema, hyperaemia and neutrophil infiltration (Figure 5B). Acetyl cholinesterase (AchE) is the necessary hydrolytic enzyme for acetyl choline in cholinergic anti-inflammatory pathway. Indeed, we noticed that LGS inhibited the activity of acetyl cholinesterase (Figure 5C), indicating that LGS attenuates acetyl choline hydrolyzation. These data together demonstrate that LGS inhibits LPS-induced ALI through cholinergic anti-inflammatory pathway.

**Figure 6:**
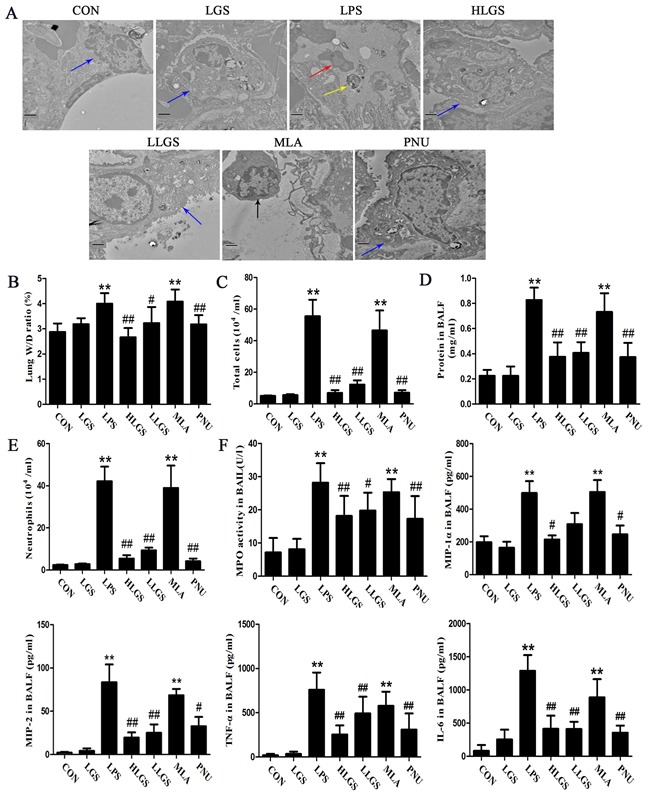
LGS inhibits edema and inflammation in LPS-induced ALI rats through cholinergic anti-inflammatory pathway LGS with or without methyllycaconitine (MLA, a α7nAchR selective inhibitor) were treated with LPS-induced ALI rats. PNU282987 (PNU), a highly selective AChR α7 agonist, was used as a positive control. **A.** MLA attenuates the influence of LGS on lung ultrastructural changes in LPS-induced ALI rats. Ultrastructures of lung tissues were observed by transmission electron microscopy (15 000 ×, Blue arrow, normal lung type II alveolar cell; Red arrow, inflammatory cell in alveolar spaces; Yellow arrow, osmilphilic multilamellar body in alveolar spaces; Black arrow, lung type II alveolar cell in alveolar spaces). **B.** MLA reduces the decrease of the ratio of lung wet weight (W) and dry weight (D) induced by LGS in ALI rats. At 8 h after ALI, lungs were separated, and weighing to get the lung W/D ratio. BALF was obtained after the sacrifice of rats to calculate the total protein concentration (**C**), total cells count (**D**) and neutrophils count (**E**). **F.** MLA attenuates the regulatory activity of LGS on inflammatory factors. After rats were sacrificed, the BALF was obtained to measure the levels of MPO, MIP-1α, MIP-2, TNF-α and IL-6 by ELISA. **P<0.01 *versus* control, ^#^*P*<0.05, and ^##^*P*<0.01 *versus* LPS, one-way ANOVA, *post hoc* comparisons, Turkey, Cloumns, mean; error bar, S.D.

Lung type II alveolar cell is regard as the protective barrier of lung and osmilphilic multilamellar body is a key structure in the type II alveolar cell to maintain cellular homeostasis [23, 24]. As shown in Figure 6A, normal lung type II alveolar cell (blue arrow) was exerted in the CON and LGS groups. Osmilphilic multilamellar body (yellow arrow) was excluded from lung type II alveolar cells in the LPS group and the lung type II alveolar cell (black arrow) was removed from the basement membrane in the MLA group, indicating the destroy of lung type II alveolar cells. It also found that inflammatory cells (red arrow) were existed in alveolar spaces in the LPS group. However, after LGS treatment, inflammatory cells were decreased and the damage on lung type II alveolar cells was inhibited, suggesting that LGS can suppress edema and inflammation. Moreover, LGS could down-regulate the oedematous indicators, including lung W/D ratio (Figure 6B), total protein (Figure 6C), total cells (Figure 6D) and neutrophils (Figure 6E) which were similar as PNU treatment. In addition, LGS attenuated inflammatory-associated factors, such as MPO, MIP-1α, MIP-2, TNF-α and IL-6 in BALF in LPS-induced ALI (Figure 6F). However, these effects of LGS could be counteracted by MLA. Taken together, our results indicate that LGS suppresses edema and inflammation in LPS-induced ALI rats through cholinergic anti-inflammatory pathway.

**Figure 7:**
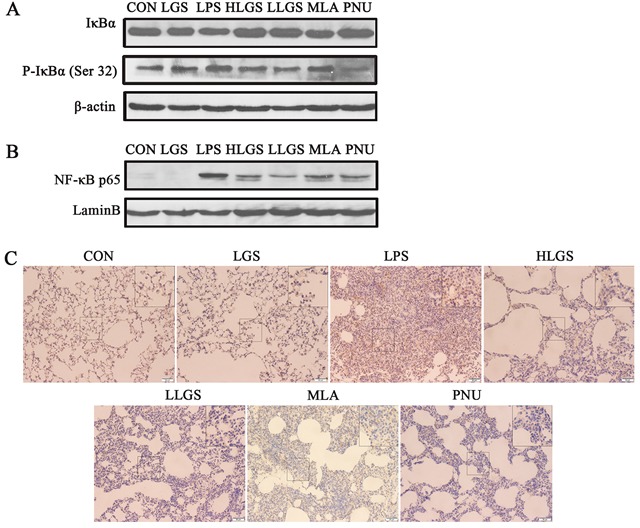
LGS inhibits the activation of NF-κB pathway in LPS-induced ALI rats through cholinergic anti-inflammatory pathway **A.** MLA counteracts the inhibitory effects of LGS on the degradation and phosphorylation of IκBα in LPS-induced ALI rats. The lung tissues were removed and homogenated after the sacrifice of rats. The total protein was used to detect the expressions of IκBα and p-IκBα (Ser32) by western blotting. **B.-C.** MLA attenuates the inhibitory effects of LGS on the translocation of NF-κB p65 from cytoplasm to nuclear. The lung tissues were removed and homogenated after the sacrifice of rats. The nuclear protein was used to detect the expression of NF-κB p65 by western blotting (B). Lungs were fixated, embeded in paraffin and sectioned at 5 μm thickness. Lung sections were stained by immunohistochemistry (C).

We further analyzed the expression of IκBα and nuclear translocation of NF-κB p65 in ALI rats. As shown in Figure 7A, LGS suppressed the degradation and phosphorylation of IκBα while MLA treatment counteracted this phenomenon. MLA also attenuated the inhibitory effects of LGS on the translocation of NF-κB p65 from cytoplasm to nuclear (Figure 7B). Similar results were observed by immunohistochemistry (Figure 7C). These results reveal that LGS inhibits the activation of NF-κB pathway in LPS-induced ALI rats through cholinergic anti-inflammatory pathway.

## DISCUSSION

Acute lung injury (ALI) is an acute respiratory failure disease with high mortality rate of 40 ~ 60 %, resulting from many severe diseases, such as sepsis pneumonia and acid aspiration [25]. Until now, effective therapeutic schedule with less side-effect has not available. The characteristics of ALI include non-cardiogenic pulmonary edema, hypoxemia, neutrophil infiltration and disrupted lung mechanics which are related to inflammation [26-28]. Accordingly, the inhibition of inflammatory response is pivotal in the progression of ALI. Clinical observation found that LGS could depress ALI [15]. Here we showed that LGS not only suppressed LPS-stimulated inflammation *in vitro*, but also inhibited LPS-induced ALI *in vivo*.

The nuclear transcription factor NF-κB is a potential positive regulator to stimulate the release of pro-inflammatory cytokines in inflammation [29]. Our present data indicated that LGS suppressed the degradation and phosphorylation of IκBα and the transactivational activity of NF-κB p65 in LPS-stimulated cell and rat models, suggesting that NF-κB pathway was involved in the anti-inflammatory effects of LGS.

Cholinergic anti-inflammatory pathway has been well known as a central nervous system suppressing peripheral inflammatory responses. There is a family of nicotinic acetylcholine receptors in this pathway and 16 different subunits have been identified, including α 1-7, α 9-10, β 1-4, γ, δ and ε [30-32]. Among them, α7 subtype expresses on macrophages and plays an important role in cholinergic anti-inflammatory pathway [5]. Studies in animal-models under the experimental conditions of sepsis, shock, or in rheumatoid arthritis patients have demonstrated that nicotine could inhibit the production of pro-inflammatory cytokines through α7nAchR [5, 6, 33, 34]. In present study, we found that LGS stimulated the expression of α7nAchR in LPS-induced macrophage cells and inflammatory responses were attenuated after α7nAchR abrogation, similar to previous research [22]. These data suggested that cholinergic anti-inflammatory pathway play a pivotal role in the anti-inflammatory effects of LGS *in vitro* and *in vivo*.

It has been indicated that the activation of cholinergic anti-inflammatory pathway involved in the suppression of NF-κB p65 pathway. In LPS-stimulated inflammation, the selective cholinergic agonist nicotine inhibited phosphorylation of IκBα and the transactivational activity of NF-κB to exert anti-inflammatory effects [6, 35, 36]. Similarly, MLA could block the inhibitory effects on TNF-α expression and NF-κB translocation stimulated by Ach in LPS-induced in Caco-2 cells [37]. We noticed that the inhibition of α7nAchR could block the degradation and phosphorylation of IκBα and the nuclear translation activity of NF-κB p65 induced by LGS. Our data implied that the activation of cholinergic anti-inflammatory pathway inhibited NF-κB pathway in the anti-inflammatory effects of LGS.

**Figure 8:**
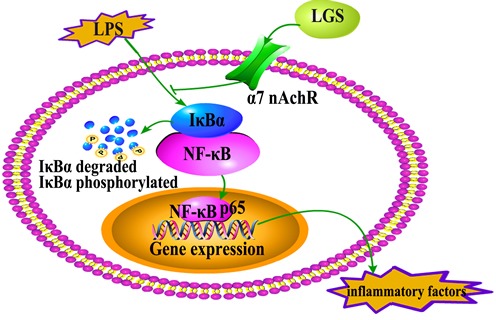
The speculated anti-inflammatory network of LGS ┴ indicates an inhibitory effect. LGS exerts anti-inflammatory effects and suppresses the degradation and phosphorylation of IκBα and the translocation of NF-κB p65 from cytoplasm to nuclear as well as the release of inflammatory factors induced by LPS through cholinergic anti-inflammatory pathway *in vitro* and *in vivo*.

In summary, we first found that LGS has significantly anti-inflammatory effects *in vitro* and *in vivo*. We elucidated the underlying mechanism that involves stimulating α7nAchR to suppress NF-κB pathway (Figure 8). Our study provides a rationale for the clinical application of LGS as an anti-inflammatory agent and supports the critical role of cholinergic anti-inflammatory pathway in inflammation, especially α7nAchR as an important anti-inflammatory target. However, LGS is a formula with multiple components, which inevitably leads to the complexity of pharmacological effects and molecular mechanisms. Therefore, to explore the specific anti-inflammatory component(s) of LGS is urgently needed.

## MATERIALS AND METHODS

### Reagents

The herbs, *Forsythia suspense* (Thunb.) Vahl, *Rheum palmatum* L., *Scutellaria baicalensis* Georgi, *Gardenia jasminoides* Ellis., *Glycyrrhiza uralensis* Fisch., *Mentha haplocalyx* Briq. and *Natrii Sulfas*. were purchased from Kangmei (Guangzhou, China). Antibodies against NF-κB p65, p-IκBα (Ser32) and IκBα were obtained from Cell Signaling Technology (Danvers, MA). LaminB, β-actin and α7nAchR antibodies were from Santa Cruz (Santa Cruz, CA). Alexa Fluor 488 secondary antibody and Lipofectamine 3000 transfection reagent were purchased from Invitrogen (Grand Island, NY). IL-6 and TNF-α ELISA kits were obtained from Dakewei (Beijing, China). Myeloperoxidase (MPO) and acetyl cholinesterase kits were purchased from Nanjing Jiancheng Bioengineering Institute (Nanjing, China) and DAB substrate kit was from Solarbio science & technology (Beijing, China) and α7nAchR small interfering RNA (siRNA) kit was from GenePharma (Shanghai, China). Nuclear and cytoplasmic protein extraction kit was purchased from Beyotime (Jiangsu, China). BCA protein assay kit and LightShift chemiluminescent EMSA kit were from Thermo Scientific (Rockford, IL). All the other reagents were purchased from Sigma Chemicals (St. Louis, MO).

LGS was prepared as described before and fingerprint analysis was performed by HPLC-DAD [38]. The freeze-dried power of LGS was dissolved in DMEM at a concentration of 20 mg/ml and stored at −20°C.

### Animals

Male Wistar rats (180 – 220g), 6 – 8 weeks old, were purchased from the Center of Experimental Animals of Southern Medical University (Guangzhou, Guangdong, China). The rats were housed in climate-controlled quarters (22 – 26°C at 40 – 70% humidity) with a 12 h light/dark cycle and free access to food and water. All experiments were performed in accordance with the National Institutes of Health guide for the care and use of laboratory animals approved by the ethical committee for the experimental use of animals at Southern Medical University (2015-002).

### Grouping and Modeling

Fifty-six rats were randomly divided into seven groups (n = 8/group): control (CON), LGS, LPS, high dose of LGS (HLGS), low dose of LGS (LLGS), methyllycaconitine (MLA, a α7nAchR selective inhibitor) and PNU282987 (PNU, a selective α7nAChR agonist). Group CON was received normal saline (1 ml/day intragastric) as the vehicle control and group LGS was treated with LGS (30 g/kg/day intragastric). Group HLGS and group LLGS were administered with a high dosage of LGS (30 g/kg/day intragastric) or low dosage of LGS (7.5 g/kg/day intragastric) [4]. Group MLA was received MLA (3 mg/kg/day intravenous) for 10 min before LGS intragastrical administration (30 g/kg/day) [19]. Group PNU was treated with PNU (2.4 mg/kg intraperitoneal) once for 20 min before the intratracheal instillation of LPS [13]. After 5 days, all groups except group CON and LGS were treated with of LPS (5 mg/kg, intratracheal) to replicate the experimental model of ALI [18]. The rats were anesthetized with 10 % (*w*/*v*) pentobarbital sodium solution to obtain BALF samples and lung tissues were removed after sacrifice for further experiments

### Cell Culture

Murine macrophage cell line RAW 264.7 was purchased from Cell Bank of the Chinese Academy of Sciences (Shanghai, China). Cells were cultured in DMEM containing 10 % (*v*/*v*) fetal bovine serum (invitrogen) and 1 % (*v*/*v*) penicillin/streptomycin (Gibco) at 37°C in a humidified incubator with 5 % CO_2_.

### Cell viability assay

Cell viability assay was performed by 3-(4, 5-dimethylthiazol-2-yl)-2, 5-diphenyltetrazolium bromide (MTT) assay. RAW 264.7 cells were cultured in a 96-well plate with the density of 5 × 10^4^ cells/ml for 24 h. Then, cells were treated with various concentrations of LGS in the presence or absence of LPS (1 μg/ml) for another 24 h. 30 μl of MTT (5 mg/ml) was added to incubate for 4 h at 37°C. The supernatant was discarded and the formazan crystal was dissolved with DMSO (100 μl/well). The absorbance was measured at 570 nm using a microplate reader (Thermo Scientific) [39].

### Enzyme-linked immunosorbent assay (ELISA)

Levels of IL-6 and TNF-α in cell supernatants and BALF were quantified with ELISA kits according to the manufacturers' instructions.

### Confocal Microscopy

RAW264.7 cells were seeded in a glass bottom dish and exposed to LGS for 24 h followed by stimulation with LPS for 2 h. Cells were rinsed with phosphate-buffered saline (PBS, PH 7.2) for three times, fixed with 4 % paraformaldehyde for 15 min, permeabilized with 0.2 % Triton X-100 for 15 min at room temperature (RT). After blocking with 5 % bovine serum albumin in PBS for 1 h, cells were incubated with NF-κB p65 antibody at 4°C overnight. Alexa Fluor 488 secondary antibody was used in dark for 1 h at 37°C before stained with 4′, 6-diamidino-2-phenylindole (DAPI) for 5 min to visualize the nuclei. Fluorescent images were photographed using a confocal microscope (Nikon) [40].

### Transient transfection with siRNA

RAW264.7 cells were seeded in a 6-well plate at 40 % confluence overnight. The α7nAchR siRNA (120 nM) was transfected into cells with Lipofectamine 3000 transfection reagent for 24 h in OPTI-MEM (Gibco) according to the manufacturer's instruction and western blotting was further conducted to confirm the silence effects.

### Preparation of total and nuclear proteins

For total protein extraction, the harvested cells or lung tissue (~ 20 mg) were lysed in lysis buffer (50 mM Tris, pH 7.5, 1% Triton X-100, 150 mM NaCl, 1 mM EDTA, 1 mM PMSF, 1mM Na_3_VO_4_, 1 mM dithiothreitol, 1 mM phosphatase inhibitor) for 30 min on ice and vortexed 15 s with 10 min interval. Samples were centrifugated at 12 000 rpm at 4°C for 15 min and the supernatants were harvested and stored at −80°C. Nuclear extract was prepared according to the manufacturers' instructions of nuclear and cytoplasmic protein extraction kit. Protein concentrations were determined with a BCA protein assay kit [39].

### Western blotting

Protein expression levels were determined by western blotting analysis. Equal amount of protein samples were separated on a 10% SDS-polyacrylamide gel electrophoresis and transferred onto a PVDF membrane (Bio-Rad). After blocking with 5% skim milk in Tris-buffered saline containing 0.1% Tween-20 (TBS-T) for 1 h, membrane was incubated with primary antibody overnight at 4°C, and probed with appropriate secondary antibody conjugated with horseradish peroxidase (Cell signaling Technology) for 1 h at RT. Bolts were detected using enhanced chamiluminescence (ECL) reagents (Bio-Rad, Hercules, CA) [39].

### Electrophoretic mobility shift assay (EMSA)

Nuclear extracts were prepared as previously described. The biotin-labeled and unlabeled double-stranded oligonucleotides containing a consensus NF-κB sequence 5′-AGT TGA GGG GAC TTT CCC AGG-3′ and its complementary strand (Beyotime) were used as the probe. EMSA was performed according to the manufacturer's protocol.

### Acetyl cholinesterase activity assay

Levels of acetyl cholinesterase activity in BALF were quantified with acetyl cholinesterase kit according to the manufacturers' protocol.

### Histopathological and immunohistochemical observation of Lung

One lobe of right lung tissues was fixed with 4 % (*w*/*v*) paraformaldehyde for 24 h. Samples were dehydrated in graded ethanol and embedded in paraffin. Some sections with 5-μm thick were stained with hematoxilin and eosin (H & E) and observed by light microscopy (Olympus IX 53). The other lung slides were incubated in citrate antigen retrieval solution (Boster) for 10 minutes at 95°C and cooled to RT. Sections were washed and incubated in 0.3 % H_2_O_2_ to quench endogenous peroxidase activity for 10 min before blocking for 2 h with 10% BSA in PBS at RT. Then, slides were incubated with NF-κB p65 antibody (1 : 800) overnight at 4°C and secondary antibody conjugated with horseradish peroxidase (1 : 1000) for 45 min at 37°C. Specific labeling was performed with DAB substrate kit and specimens were counterstained with hematoxilin before being observed by light microscopy (Olympus IX 53).

### Transmission electron microscope ultrastructural observation

After LGS treatment, lung specimens were immediately fixed in PBS containing 2.5% glutaraldehyde and stored at 4°C overnight. Tissue samples were postfixed in osmium tetroxide and embedded in Spurr's resin. Ultrathin sections (50 nm) were collected and stained with 1% uranyl acetate and 0.2 % lead citrate before observed by transmission electron microscopy (Hitachi H-7650).

### Lung wet/dry weight (W/D) ratio measurement

Lung tissues were weighed to obtain the wet weight (W) and then placed in an oven at 60°C for 72 h to obtain the dry weight (D). To assess the tissue edema, W/D ratio was calculated by the following formula: wet weight/dry weight × 100 %.

### Protein levels, cell count and MPO activity in BALF

After LPS treatment for 8 h, the rats were anesthetized with 10% (*w*/*v*) pentobarbital sodium solution and were inserted with a plastic cannula into the trachea. BALF was performed with three aliquots of 1 ml PBS instilled up to a total volume of 3 ml. BALF samples were centrifuged (3 000 rpm, 4°C) for 10 min and supernatants were stored at −80°C for subsequent analysis. The protein concentrations in BALF supernatants were measured by a BCA protein assay kit. The sediment cells were re-suspended in 50 μl PBS and total BALF cells were counted double-blindly using a hemacytometer, following leukocytes differential counting which were stained with Wright Giemsa. MPO activity in the BALF was measured with MPO determination kit according to the manufacturers' instruction. Absorbance was measured at 460 nm on a microplate reader (Thermo Scientific).

### Statistical analysis

Results are presented as the mean ± standard deviation (S.D.) from 3 independent experiments at least. The data was analyzed by Graph Pad Prism 5.0 (Graph Pad, La Jolla, CA). *P* < 0.05 was considered statistically significant.
